# Supplementary material for: Transcriptomic analyses reveal rhythmic and CLOCK-driven pathways in human skeletal muscle
Source: eLife. 2018 Apr 16;7:e34114. doi: 10.7554/eLife.34114 (PMC5902165; doi:10.7554/eLife.34114)
Supplement: Supplementary file 1. [file elife-34114-supp1.docx]

Supplementary data to:

**Transcriptomic analyses reveal rhythmic and CLOCK-driven pathways in human skeletal muscle**

Laurent Perrin ^1,2,3,4^, Ursula Loizides-Mangold ^1,2,3,4,†^, Stéphanie Chanon ^5,†^, Cédric Gobet ^6,7,†^, Nicolas Hulo ^4,8^, Laura Isenegger ^8^, Benjamin D. Weger ^6^, Eugenia Migliavacca ^6^, Aline Charpagne ^6^, James A. Betts ^9^, Jean-Philippe Walhin ^9^, Iain Templeman ^9^, Keith Stokes ^9^, Dylan Thompson ^9^, Kostas Tsintzas ^10^, Maud Robert ^11^, Cédric Howald ^4,12^, Howard Riezman ^13^, Jerome N Feige ^6,7^, Leonidas G. Karagounis ^14,15^, Jonathan D. Johnston ^16^, Emmanouil Dermitzakis ^4,12^, Frédéric Gachon ^6,7,ç^, Etienne Lefai ^5,ç^, Charna Dibner ^1,2,3,4,^*

^1^Division of Endocrinology, Diabetes, Hypertension and Nutrition, Department of Internal Medicine Specialties, University Hospital of Geneva, Geneva, Switzerland;

^2^Department of Cell Physiology and Metabolism, Faculty of Medicine, University of Geneva;

^3^Diabetes Center, Faculty of Medicine, University of Geneva;

^4^Institute of Genetics and Genomics in Geneva (iGE3), Geneva, Switzerland;

^5^CarMeN Laboratory, INSERM U1060, INRA 1397, University Lyon 1, Oullins, France;

^6^Nestlé Institute of Health Sciences, Lausanne, Switzerland;

^7^School of Life Sciences, Ecole Polytechnique Fédérale de Lausanne, Lausanne, Switzerland;

^8^Service for Biomathematical and Biostatistical Analyses; Section of Biology, University of Geneva, Geneva, Switzerland;

^9^Department for Health, University of Bath, Bath, United Kingdom;

^10^MRC/ARUK Centre for Musculoskeletal Ageing, School of Life Sciences, University of Nottingham, NG7 2UH, United Kingdom;

^11^Department of Digestive and Bariatric Surgery, Edouard Herriot University Hospital, Lyon 1 University, France;

^12^Department of Genetic Medicine and Development, Faculty of Medicine, University of Geneva, Geneva, Switzerland;

^13^Department of Biochemistry, NCCR Chemical Biology, University of Geneva, Geneva, Switzerland;

^14^Experimental Myology and Integrative Biology Research Cluster, Faculty of Sport and Health Sciences, University of St Mark and St John, Plymouth, United Kingdom;

^15^Institute of Nutritional Science, Nestlé Research Centre, Lausanne, Switzerland;

^16^Faculty of Health and Medical Sciences, University of Surrey, Guildford, United Kingdom.

^†^These authors contributed equally to this work

^ç^These authors contributed equally to this work

***CORRESPONDING AUTHOR**

Charna Dibner

Faculty of Medicine, University of Geneva

D05.2147c Rue Michel-Servet, 1

CH-1211 Geneva 4, Switzerland

Phone: +41 22 3795934

**SUPPLEMENTARY TABLES**

**Table S1.** **Characteristics of donors for skeletal muscle biopsies**

| Donor n° | Sex | Age (years) | BMI (kg/m2) | Muscle | Used for |  |
| --- | --- | --- | --- | --- | --- | --- |
| I | M | 22 | 28.3 | *Vastus lateralis* | RNA-seq | *In vivo* |
| II | F | 37 | 28.3 | *Vastus lateralis* |  |  |
| III | M | 27 | 20.4 | *Vastus lateralis* |  |  |
| IV | M | 33 | 23.6 | *Vastus lateralis* |  |  |
| V | M | 54 | 25.6 | *Vastus lateralis* |  |  |
| VI | M | 24 | 24.6 | *Vastus lateralis* |  |  |
| VII | M | 25 | 23.8 | *Vastus lateralis* |  |  |
| VIII | M | 30 | 20.3 | *Vastus lateralis* |  |  |
| IX | M | 22 | 23.2 | *Vastus lateralis* |  |  |
| X | M | 25 | 23.4 | *Vastus lateralis* |  |  |
| Mean | M=9, F=1 | 30 ± 10 | 24.1 ± 2.7 |  |  |  |
| 1 | M | 62 | 24.3 | *Gluteus maximus* | RNA-seq | *In vitro* |
| 2 | M | 60 | 24 | *Gluteus maximus* |  |  |
| Mean | M=2 | 61 ± 1 | 24.15 ± 0.21 |  |  |  |
| 3 | F | 66 | 24 | *Gluteus maximus* | Glucose uptake  &  Western blot | *In vitro* |
| 4 | F | 65 | 22 | *Gluteus maximus* |  |  |
| 5 | M | 70 | 30.1 | *Gluteus maximus* |  |  |
| 6 | M | 72 | 26.5 | *Gluteus maximus* |  |  |
| 7 | F | 77 | 25.6 | *Gluteus maximus* |  |  |
| Mean | M=2, F=3 | 70 ± 4 | 25.64 ± 3.02 |  |  |  |
| 7 | F | 77 | 25.6 | *Gluteus maximus* | Lipidomics | *In vitro* |
| 8 | M | 57 | 26.3 | *Gluteus maximus* |  |  |
| 9 | F | 65 | 22.77 | *Gluteus maximus* |  |  |
| 10 | F | 53 | 22.41 | *Gluteus maximus* |  |  |
| Mean | M=1, F=3 | 63 ± 10 | 24.27 ± 1.97 |  |  |  |

M, male; F, female.

Donors I-X, data are mean ± SD, N=10.

Donors 1-2, data are mean ± SD, N=2.

Donors 3-7, data are mean ± SD, N=5.

Donors 7-10, data are mean ± SD, N=4.

**Table S2.** Selected genes involved in cargo trafficking and GLUT4 regulation that have altered expression upon siCLOCK

| Function | Name | % of change | p-value | FDR |
| --- | --- | --- | --- | --- |
| Cargo trafficking | *ARFGAP2* | 8.60 | 9.73E-06 | 4.11E-04 |
|  | *CAMSAP2* | -8.30 | 1.52E-04 | 4.03E-03 |
|  | *EPN2* | -10.05 | 3.49E-04 | 7.98E-03 |
|  | *FNBP1* (*FNBP17*) | -10.20 | 7.18E-04 | 1.42E-02 |
|  | *GBF1* | -7.85 | 3.15E-04 | 7.35E-03 |
|  | *HIP1* | 11.55 | 2.41E-04 | 5.96E-03 |
|  | *KIF13A* | -23.56 | 1.22E-27 | 1.14E-24 |
|  | *PACSIN3* | 18.17 | 7.65E-07 | 4.47E-05 |
|  | *PAFAH1B1* (*LYS1*) | -6.31 | 3.82E-04 | 8.58E-03 |
|  | *STX6* | -13.17 | 1.71E-10 | 2.24E-08 |
|  | *SYT1* | 21.45 | 1.20E-03 | 2.11E-02 |
|  | *VAMP3* | -26.89 | 1.38E-27 | 1.22E-24 |
|  | *VAMP4* | -16.92 | 2.17E-09 | 2.22E-07 |
|  | *VAMP8* | 15.77 | 1.21E-03 | 2.12E-02 |
|  | *VPS36* (*VSP36*) | -15.48 | 9.14E-11 | 1.28E-08 |
| Regulation of GLUT4 translocation | *14-3-3θ* (*YWHAQ*) | -14.70 | 1.70E-07 | 1.22E-05 |
|  | *CALM1* [1+3] | -14.20 | 3.72E-06 | 1.80E-04 |
|  | *CAV3* [1] | 18.72 | 6.26E-10 | 7.35E-08 |
|  | *EXOC3L4* | -22.87 | 3.54E-04 | 8.08E-03 |
|  | *MAPKAP1* (*mSIN1*) | 8.90 | 3.07E-08 | 2.53E-06 |
|  | *MEF2A* | -28.09 | 3.25E-13 | 7.00E-11 |
|  | *NAPB* | -9.99 | 1.17E-04 | 3.24E-03 |
|  | *NAPG* | 10.01 | 2.70E-03 | 3.81E-02 |
|  | *NSF* | 13.26 | 1.94E-08 | 1.67E-06 |
|  | *PDPK1* (*PDK1*) | -9.01 | 3.81E-08 | 3.10E-06 |
|  | *RAB11FIP4* | -17.90 | 6.95E-05 | 2.13E-03 |
|  | *RAB35* | -22.93 | 3.63E-23 | 2.18E-20 |
|  | *RAB5B* | 6.46 | 1.33E-04 | 3.63E-03 |
|  | *RALGAPA2* (*RGC2*) | -14.26 | 2.29E-04 | 5.72E-03 |
|  | *TBC1D4* (*AS160*) | -8.38 | 1.99E-03 | 3.05E-02 |
|  | *TBC1D13* | -5.94 | 1.78E-03 | 2.82E-02 |
|  | *TBC1D16* | -14.91 | 3.80E-05 | 1.29E-03 |
|  | *TPM3* [1] | 10.01 | 1.29E-03 | 2.23E-02 |
|  | *VPS45* (*VSP45*) | -7.26 | 1.41E-04 | 3.81E-03 |

**Bold**: gene classified as circadian (models 1 to 4).

Underscore: genes also found in regulation of GLUT4 translocation.

[n]: number corresponding to the functional group listed in Table S7.

*: gene with a FDR>0.05.

**Table S3.** Selected genes involved in secretion that have altered mRNA expression upon siCLOCK

| Name | % of change | p-value | FDR |
| --- | --- | --- | --- |
| *ABCB6* | -13.21 | 1.37E-06 | 7.37E-05 |
| *ABCC5* | -16.48 | 1.32E-05 | 5.29E-04 |
| *ATP1B4* | 8.85 | 9.91E-04 | 1.84E-02 |
| *ATP5G2* | -32.87 | 1.99E-42 | 4.17E-39 |
| *ATP5G2P4* | -37.85 | 9.18E-11 | 1.28E-08 |
| *ATP7B* | 28.54 | 1.98E-12 | 3.74E-10 |
| *CACNA1E* | 28.83 | 7.57E-11 | 1.09E-08 |
| *DNAH10OS* | 17.35 | 3.08E-03 | 4.18E-02 |
| *DYNLL2* | 9.72 | 4.32E-05 | 1.45E-03 |
| *ESYT2* | -9.91 | 7.04E-05 | 2.15E-03 |
| *KCNB1* | 27.57 | 6.79E-15 | 1.93E-12 |
| *KIF1A* | 17.72 | 2.94E-03 | 4.05E-02 |
| *PCSK1* | -18.24 | 3.54E-03 | 4.62E-02 |
| *RAB11FIP1* | 19.46 | 2.53E-04 | 6.20E-03 |
| *RAB15* | 23.31 | 1.57E-06 | 8.39E-05 |
| *RABGAP1* | -10.20 | 2.06E-06 | 1.07E-04 |
| *SEC61A1* | -15.71 | 3.08E-07 | 2.02E-05 |
| *SLC1A3* | -16.10 | 1.97E-04 | 5.05E-03 |
| *SLC22A17* | -13.13 | 8.05E-06 | 3.45E-04 |
| *SLC22A31* | -29.20 | 4.80E-07 | 3.02E-05 |
| *SLC24A2* | -12.60 | 5.19E-04 | 1.10E-02 |
| *SLC25A12* | -8.48 | 5.97E-04 | 1.23E-02 |
| *SLC25A3* | 4.83 | 3.42E-03 | 4.50E-02 |
| *SLC25A44* | -18.83 | 3.00E-09 | 2.96E-07 |
| *SLC26A7* | 36.77 | 3.84E-04 | 8.60E-03 |
| *SLC28A3* | 24.73 | 1.37E-03 | 2.35E-02 |
| *SLC2A3P1* | 11.18 | 4.86E-05 | 1.60E-03 |
| *SLC30A6* | -17.80 | 1.74E-09 | 1.82E-07 |
| *SLC39A14* | -8.51 | 1.77E-03 | 2.80E-02 |
| *SLC46A1* | -11.64 | 1.49E-03 | 2.48E-02 |
| *SLC47A2* | -24.49 | 3.40E-03 | 4.48E-02 |
| *SLC4A4* | -11.39 | 3.81E-03 | 4.87E-02 |
| *SLC6A6* | 12.05 | 1.11E-03 | 1.99E-02 |
| *SLC7A7* | 11.21 | 1.62E-03 | 2.63E-02 |
| *SLC7A8* | -22.71 | 1.76E-03 | 2.80E-02 |
| *SLC9A6* | -12.59 | 1.23E-09 | 1.35E-07 |
| *SLC9B2* | -10.72 | 1.53E-05 | 5.91E-04 |
| *SLCO5A1* | -25.08 | 2.85E-13 | 6.30E-11 |
| *SYT6* | -28.27 | 6.20E-07 | 3.73E-05 |
| *TBC1D12* | 10.80 | 2.44E-03 | 3.53E-02 |
| *TBC1D19* | -8.62 | 2.99E-03 | 4.09E-02 |

**Table S4.** Enrichment of biological processes based on gene ontology (GO) term analysis

| Category | PANTHER GO-Slim Biological Process | Fold enrichment | p-value |
| --- | --- | --- | --- |
| Downregulated | transcription, DNA-dependent (GO:0006351) | 2.48 | 3.07E-05 |
| Downregulated | _└_ RNA metabolic process (GO:0016070) | 2.03 | 7.60E-06 |
| Downregulated | _└_ nucleobase-containing compound  metabolic process (GO:0006139) | 1.46 | 8.72E-04 |
| Downregulated | _└_ primary metabolic process (GO:0044238) | 1.31 | 7.33E-04 |
| Downregulated | _└_ **metabolic process (GO:0008152)** | 1.26 | 4.09E-03 |
| Downregulated | **regulation of nucleobase-containing compound metabolic process (GO:0019219)** | 1.83 | 2.85E-05 |
| Downregulated | **biosynthetic process (GO:0009058)** | 1.7 | 4.51E-04 |
| Downregulated | **nitrogen compound metabolic process (GO:0006807)** | 1.61 | 1.90E-04 |
| Downregulated | **cellular process (GO:0009987)** | 1.21 | 8.15E-03 |
| Upregulated | **muscle contraction (GO:0006936)** | 4.97 | 5.06E-06 |
| Down- & upregulated | **transcription, DNA-dependent (GO:0006351)** | 1.76 | 1.61E-02 |
| Down- & upregulated | **regulation of nucleobase-containing compound metabolic process (GO:0019219)** | 1.47 | 5.41E-03 |
| Down- & upregulated | **cellular process (GO:0009987)** | 1.18 | 3.22E-04 |
| Model 1 | chromosome segregation (GO:0007059) | 14.43 | 6.60E-03 |
| Model 1 | _└_ **cellular process (GO:0009987)** | 1.79 | 2.38E-04 |
| Model 1 | **DNA metabolic process (GO:0006259)** | 5.97 | 4.21E-02 |
| Model 1 | **cell cycle (GO:0007049)** | 5.05 | 1.29E-04 |

**Bold**: main GO terms listed in Figure 2-source data 2 and in Figure 4-source data 3.

**Table S5.** Reactome pathway enrichment analysis

| Category | Reactome pathways | Fold  enrichment | p-value |
| --- | --- | --- | --- |
| Downregulated | Generic Transcription Pathway (R-HSA-212436) | 2.91 | 6.19E-13 |
| Downregulated | _└_ **Gene Expression (R-HSA-74160)** | 2.09 | 4.42E-10 |
| Downregulated | Smooth Muscle Contraction (R-HSA-445355) | 8.75 | 3.56E-02 |
| Upregulated | _└_ **Muscle contraction (R-HSA-397014)** | 3.78 | 2.23E-03 |
| Down- & upregulated | Generic Transcription Pathway (R-HSA-212436) | 2.1 | 1.90E-08 |
| Down- & upregulated | _└_ **Gene Expression (R-HSA-74160)** | 1.57 | 8.26E-05 |
| Down- & upregulated | **Cellular responses to stress (R-HSA-2262752)** | 2.1 | 2.28E-02 |
| Down- & upregulated | **Membrane Trafficking (R-HSA-199991)** | 1.83 | 4.80E-02 |
| Model 1 | Phosphorylation of Emi1 (R-HSA-176417) | > 100 | 9.48E-06 |
| Model 1 | _└_ APC/C-mediated degradation of cell cycle  proteins (R-HSA-174143) | 17.96 | 1.72E-02 |
| Model 1 | _└_ Regulation of mitotic cell cycle (R-HSA-  453276) | 17.96 | 1.72E-02 |
| Model 1 | _└_ Cell Cycle, Mitotic (R-HSA-69278) | 11.32 | 1.94E-10 |
| Model 1 | _└_ **Cell Cycle (R-HSA-1640170)** | 9.24 | 4.74E-09 |
| Model 1 | Activation of NIMA Kinases NEK9, NEK6, NEK7 (R-HSA-2980767) | > 100 | 3.24E-03 |
| Model 1 | _└_ **M Phase (R-HSA-68886)** | 10.14 | 4.78E-04 |
| Model 1 | BMAL1:CLOCK,NPAS2 activates circadian gene expression (R-HSA-1368108) | 73.54 | 4.75E-13 |
| Model 1 | _└_ **Circadian Clock (R-HSA-400253)** | 49.81 | 2.22E-11 |
| Model 1 | **Golgi Cisternae Pericentriolar Stack Reorganization (R-HSA-162658)** | 71.27 | 2.05E-02 |
| Model 1 | TP53 Regulates Transcription of Genes Involved in G2 Cell Cycle Arrest (R-HSA-6804114) | 68.63 | 7.46E-04 |
| Model 1 | _└_ **TP53 Regulates Transcription of Cell Cycle Genes (R-HSA-6791312)** | 32.17 | 1.02E-03 |
| Model 1 | Polo-like kinase mediated events (R-HSA-156711) | 57.91 | 3.79E-02 |
| Model 1 | _└_ G2/M Transition (R-HSA-69275) | 11.75 | 4.27E-03 |
| Model 1 | _└_ **Mitotic G2-G2/M phases (R-HSA-453274)** | 11.62 | 4.58E-03 |
| Model 1 | G0 and Early G1 (R-HSA-1538133) | 49.42 | 2.73E-03 |
| Model 1 | _└_ **Mitotic G1-G1/S phases (R-HSA-453279)** | 17.78 | 3.39E-05 |
| Model 1 | Activation of the pre-replicative complex (R-HSA-68962) | 38.61 | 7.20E-03 |
| Model 1 | _└_ **G1/S Transition (R-HSA-69206)** | 16.11 | 3.91E-03 |
| Model 1 | Activation of ATR in response to replication stress (R-HSA-176187) | 33.39 | 1.27E-02 |
| Model 1 | _└_ G2/M Checkpoints (R-HSA-69481) | 12.44 | 1.70E-02 |
| Model 1 | _└_ **Cell Cycle Checkpoints (R-HSA-69620)** | 11.94 | 3.83E-03 |
| Model 1 | Resolution of Sister Chromatid Cohesion (R-HSA-2500257) | 21.87 | 6.91E-06 |
| Model 1 | _└_ **Mitotic Prometaphase (R-HSA-68877)** | 20.42 | 1.17E-05 |
| Model 1 | RHO GTPases Activate Formins (R-HSA-5663220) | 14.71 | 6.58E-03 |
| Model 1 | _└_ **RHO GTPase Effectors (R-HSA-195258)** | 8.28 | 4.06E-02 |
| Model 1 | **Separation of Sister Chromatids (R-HSA-2467813)** | 10.47 | 4.45E-02 |

**Bold**: main pathways listed in Figure 2-source data 1 and in Figure 4-source data 2.

**Table S6.** Effect of siCLOCK on the amplitude of selected circadian genes from models 1-4

| Name | siControl^a^ | siCLOCK^a^ | Amplitude | p-value^b^ | Significance | Model |
| --- | --- | --- | --- | --- | --- | --- |
| *ALDH1A3* | -0.018 | -0.175 | Reduced | 4.26E-02 | * | 1 |
| *CAMKK1* | 0.370 | 0.229 | Reduced | 1.35E-02 | * |  |
| *CRY1* | 0.092 | -0.069 | Reduced | 1.41E-02 | * |  |
| ***KIF2C*** | **-0.053** | **0.058** | **Increased** | **1.54E-03** | ****** |  |
| *LPPR4* | 0.055 | -0.106 | Reduced | 3.87E-02 | * |  |
| *PER1* | 0.267 | 0.072 | Reduced | 3.55E-02 | * |  |
| *PER2* | 0.211 | -0.005 | Reduced | 3.48E-02 | * |  |
| *PER3* | 0.398 | 0.211 | Reduced | 9.75E-03 | ** |  |
| *SERPINE1* | 0.079 | -0.045 | Reduced | 4.89E-03 | ** |  |
| *TEF* | 0.414 | 0.293 | Reduced | 4.62E-02 | * |  |
| *TLR3* | -0.022 | -0.055 | Reduced | 2.88E-02 | * |  |
| *BHLHE40* | -0.115 | -0.280 | Reduced | 2.64E-02 | * | 2 |
| *LINC00857* | -0.066 | -0.273 | Reduced | 2.54E-02 | * |  |
| ***NPR3*** | **-0.216** | **-0.144** | **Increased** | **1.59E-03** | ****** |  |
| ***H2AFX*** | **-0.309** | **-0.191** | **Increased** | **3.63E-03** | ****** | 3 |
| *NUDT6* | 0.127 | 0.068 | Reduced | 4.32E-02 | * |  |
| *RMI1* | -0.022 | -0.102 | Reduced | 7.60E-04 | *** |  |
| *EFNA5* | 0.053 | -0.258 | Reduced | 4.87E-02 | * | 4 |
| *FOXC1* | -0.097 | -0.283 | Reduced | 3.23E-03 | ** |  |
| *PDGFC* | -0.126 | -0.284 | Reduced | 6.72E-03 | ** |  |
| *RP11-61A14.2* | 0.076 | -0.135 | Reduced | 1.17E-02 | * |  |
| *ZFP36* | -0.234 | -0.464 | Reduced | 2.64E-02 | * |  |

^a^: Data represent the mean amplitude (in log_10_) of the two cell lines per condition.

^b^: Paired T-test, N=2 per condition, p-value (*)<0.05, (**)<0.01, (***)<0.001.

**Table S7.** Selected genes with modified expression involved in muscle function, metabolism, cell cycle and apoptosis

| Function | Name | % of change |
| --- | --- | --- |
| 1. Calcium flux and muscle contraction | *ATP1B1* | -13.99 |
|  | *ATP2A1* (*SERCA1*) [3] | -32.55 |
|  | *CACNA2D1* | 7.02 |
|  | *CACNG1* | 12.17 |
|  | *CALD1* | 10.63 |
|  | *CAMK2A* | -13.57 |
|  | *CAMK2G* | -11.04 |
|  | *CASQ2* [2] | 11.71 |
|  | *KAT2B* | -33.67 |
|  | *KCND3* | 26.98 |
|  | *KCNE1L* (*KCNE5*) | 27.34 |
|  | *MYH3* [2] | 22.78 |
|  | *MYL12A* | 15.68 |
|  | *MYL2* [2] | 33.51 |
|  | *MYL3* [2] | 51.93 |
|  | *MYL9* | 14.07 |
|  | *MYLK* | 22.36 |
|  | *MYLPF* [3] | 11.45 |
|  | *PVALB* | 23.06 |
|  | *SCN1B* | -14.84 |
|  | *SCN5A* | 12.51 |
|  | *SLC8A3* | -22.17 |
|  | *SRL* | 16.75 |
|  | *TNNC1* [2] | 21.46 |
|  | *TNNC2* [3] | 10.96 |
|  | *TNNI1* [2] | 10.40 |
|  | *VCL* [3] | -12.71 |
| 2. Slow twitch gene expression | *CSRP2* | 24.53 |
|  | *CSRP3* | 37.59 |
|  | *FHL1* | 13.04 |
|  | *MYOZ2* | 23.32 |
|  | *XIRP1* | 20.97 |
| 3. Fast twitch gene expression | *FHL3* | -11.04 |
|  | *MYH2* | 12.40 |
|  | *MYOG* | 21.04 |
|  | *PBX2* | -9.41 |
|  | *PDLIM1* | 10.51 |
| 4. Muscle energy provision | *CKM* | 13.97 |
|  | *EGLN1* [5] | -10.82 |
|  | *FABP3** | 17.08 |
|  | *GYS1* | -10.74 |
|  | *PYGL* | 11.59 |
| 5. HIF1α signaling | *VEGFA* | -18.04 |
|  | *VHL* | -13.82 |
| 6. Metabolic gene expression | *ACOT12* | 68.74 |
|  | *AGPAT4** | -10.67 |
|  | *AKT3* | -8.20 |
|  | *CD36** | 25.20 |
|  | *COX6A2* | 10.14 |
|  | *NDUFA7* | -34.98 |
|  | *NDUFS2* | 6.40 |
|  | *PGM5* | 24.34 |
|  | *PHKA1* | -11.92 |
|  | *PRKAG2* | 12.96 |
|  | *PRKAG3* | -13.85 |
|  | *SDHC* | 5.95 |
|  | *UQCR11* | -10.62 |
| 7. Protein glycosylation | *AGER* | -19.43 |
|  | *BCAN* | 39.64 |
|  | *C1GALT1* | -16.91 |
|  | *CD34* | 38.98 |
|  | *DPAGT1* | -8.37 |
|  | *DPH3* | 9.37 |
|  | *EXTL3* | 8.89 |
|  | *FAM20B* | 8.26 |
|  | *GALNT12* | -9.70 |
|  | *GALNT15* | -33.29 |
|  | *GALNT7* | -24.63 |
|  | ***GFPT2*** | **-26.59** |
|  | *GNPTAB** | -11.12 |
|  | *GPC1* | -29.71 |
|  | *IMPG2* | 16.72 |
|  | *LYSMD2* | -11.71 |
|  | *MPI* | 11.97 |
|  | *PRG4* | -25.46 |
|  | *SGCD* | -13.47 |
|  | *VCAN* | -14.93 |
|  | *VCAN-AS1* | -14.74 |
| 8. Polyamine metabolism | *ADC* | -10.38 |
|  | *OAZ2* | 6.89 |
|  | *SAT1* | -16.06 |
| 9. GPI anchor/Lipid glycosylation | *ART1* | 126.17 |
|  | *B3GAT2* | -9.50 |
|  | *B3GNT4* | -11.36 |
|  | *PIGG* | -5.72 |
|  | *PIGS* | 6.51 |
| 10. Muscle atrophy | *MSTN* | 43.11 |
|  | *TRIM63* | 19.58 |
| 11. Cell cycle | *CDK6* | 16.47 |
|  | *CLASP1* | -11.03 |
|  | *NONO* | 6.96 |
|  | *RB1* | 8.63 |
|  | *TEAD1* (*TEA1*) | -11.18 |
|  | *TEAD2* (*TEA2*) | 8.60 |
|  | *WEE1* | 19.23 |
| 12. Apoptosis | *APAF1* | -16.07 |
|  | *DAPK1* | -27.96 |
|  | *MYCL* [10] | -30.67 |

**Bold**: circadian genes according to our models 1-4.

[n]: function number where a gene could also belong.

*: Genes presented in Figure 2 C and E.

**Table S8.** List of common significantly affected genes upon *CLOCK* silencing between human islets and hSKM (Saini et al. 2016).

| Name | % of  change | Name | % of  change | Name | % of  change |
| --- | --- | --- | --- | --- | --- |
| *AACS* | -12.68 | *NR3C1* | -10.98 | ***CRY1*** | **34.52** |
| *ADAM10* | -21.79 | *NRP1* | -12.99 | *EZR* | 16.55 |
| *ADAR* | -8.93 | *NUDT21* | -18.05 | *MMP15* | 20.97 |
| *AIDA* | -11.19 | *OIP5-AS1* | -28.90 | *NIPA1* | 21.51 |
| *AKAP2* | -12.17 | *OSMR* | -12.46 | *PEG10* | 19.92 |
| *ANAPC15* | -22.58 | *PAFAH1B2* | -9.07 | *PODXL2* | 17.22 |
| *ANTXR2* | -23.90 | *PALM2-AKAP2* | -11.99 | *PSEN2* | 10.36 |
| *ARCN1* | -24.40 | *PCCB* | -14.84 | *PSKH1* | 27.86 |
| *ARHGEF17* | -14.28 | ***PER3*** | **-34.92** | *SGPL1* | 15.94 |
| *ATP5G2* | -32.87 | *PHLDB1* | -11.10 | *VAT1* | 10.35 |
| *BCAT1* | -11.46 | *PPIP5K2* | -19.38 |  |  |
| *BEX4* | -16.92 | *PPP2R3A* | -10.37 |  |  |
| ***BHLHE41*** | **-17.66** | *PRDX3* | -13.52 |  |  |
| *BTG2* | -16.62 | *RP11-334E6.12* | -14.25 |  |  |
| *BVES* | -24.35 | *RYBP* | -17.62 |  |  |
| *C1GALT1* | -16.91 | *SAMD8* | -20.95 |  |  |
| *C1orf51* | -45.82 | *SLC25A44* | -18.83 |  |  |
| *CCDC6* | -15.44 | *SLC30A6* | -17.80 |  |  |
| *CD46* | -14.05 | *SSR1* | -16.79 |  |  |
| *CLOCK* | -80.04 | *TEAD1* | -11.18 |  |  |
| *COL3A1* | -7.96 | ***TEF*** | **-32.96** |  |  |
| *CTDSPL* | -37.50 | *TFDP1* | -20.28 |  |  |
| *DBP* | -36.42 | *THY1* | -15.10 |  |  |
| *EIF4EBP2* | -39.43 | *TNPO1* | -7.12 |  |  |
| *FAM126B* | -18.87 | *TNS1* | -8.67 |  |  |
| *FAM160B1* | -13.19 | *TXN2* | -22.32 |  |  |
| *FAM172A* | -11.94 | *UBA1* | -8.94 |  |  |
| *FAM189B* | -13.88 | *UBE4A* | -12.66 |  |  |
| *FAM210B* | -12.29 | *UBE4B* | -8.42 |  |  |
| *FICD* | -25.80 | *USP22* | -8.44 |  |  |
| *FURIN* | -17.52 | *VCAN-AS1* | -14.74 |  |  |
| *GALNT7* | -24.63 | *VCL* | -12.71 |  |  |
| *GNAQ* | -9.93 | *VDAC1* | -13.82 |  |  |
| *GPC1* | -29.71 | *WDR83OS* | -15.73 |  |  |
| *GPR124* | -11.64 | *ZNF252P* | -27.82 |  |  |
| *KIF13A* | -23.56 | *ZNF268* | -31.67 |  |  |
| *LUM* | -10.08 | *ZNF468* | -38.36 |  |  |
| *MAGED2* | -15.95 | *ZNF664* | -10.01 |  |  |
| *MAP3K11* | -22.32 | *VAMP3* | -26.89 |  |  |
| *MAPK6* | -18.23 |  |  |  |  |
| *MAPKAPK2* | -15.72 |  |  |  |  |
| *MDFIC* | -14.52 |  |  |  |  |
| *MEF2A* | -28.09 |  |  |  |  |
| *MEX3C* | -16.20 |  |  |  |  |
| *NPC1* | -23.35 |  |  |  |  |
| ***NR1D1* (*REVERBα*)** | **-46.90** |  |  |  |  |
| ***NR1D2 (REVERB*β*)*** | **-35.56** |  |  |  |  |

**Bold**: circadian genes according to our models 1-4.

Underscore: genes also found in Table S4.

**Table S9**. Sequences of human RT-qPCR primers**.**

| Gene name | Primer sequence | |
| --- | --- | --- |
| *ARNTL/BMAL1* | Forward | 5'-CCCTTGGACCAAGGAAGTAGAA-3' |
|  | Reverse | 5'-CTTCCAGGACGTTGGCTAAAAC-3' |
| *NR1D1/REVERBα* | Forward | 5'-GATCGTGAGTCGCGGGGTCC-3' |
|  | Reverse | 5'-TGTAGGTGATGACGCCACCTGTGT-3' |
| *PER3* | Forward | 5'-CCTGGACCCTGAACATGCA-3' |
|  | Reverse | 5'-TGTGAGCCCCACGTGTTTAA-3' |
| *CLOCK* | Forward | 5'-CAAGCCACCGCAACAATT-3' |
|  | Reverse | 5'-GGATTCCCATGGAGCAACCTA-3' |
| *9S* | Forward | 5'-CTCCGGAACAAACGTGAGGT-3' |
|  | Reverse | 5'-TCCAGCTTCATCTTGCCCTC-3' |
| *HPRT* | Forward | 5'-GATTTTATCAGACTGAGGAGC-3' |
|  | Reverse | 5'-TCCAGTTAAAGTTGAGAGATC-3' |
| *Myco711* | Forward | 5'-CGCCTGAGTAGTACGTTCGC-3' |
| *Myco712* | Forward | 5'-CGCCTGGGTAGTACATTCGC-3' |
| *Myco713* | Forward | 5'-CGCCTGAGTAGTATGCTCGC-3' |
| *Myco715* | Forward | 5'-CGCCTGAGTAGTACGTACGC-3' |
| *Myco716* | Forward | 5'-TGCCTGAGTAGTACATTCGC-3' |
| *Myco717* | Forward | 5'-TGCCTGGGTAGTACATTCGC-3' |
| *Myco709* | Reverse | 5'-GCGGTGTGTACAAGACCCGA-3' |
| *Myco710* | Reverse | 5'-GCGGTGTGTACAAAACCCGA-3' |
| *Myco714* | Reverse | 5'-GCGGTGTGTACAAACCCCGA-3' |
