# Supplementary material for: Transcriptomic analyses reveal rhythmic and CLOCK-driven pathways in human skeletal muscle
Source: eLife. 2018 Apr 16;7:e34114. doi: 10.7554/eLife.34114 (PMC5902165; doi:10.7554/eLife.34114)
Supplement: Transparent reporting form [file elife-34114-transrepform.pdf]

eLIFE
1st Floor
24 Hills Road
Cambridge CB2 1JP, UK
P 01223 855340
W elifesciences.org
T @elife

eLife's transparent reporting form

We encourage authors to provide detailed information *within their submission* to facilitate the interpretation and replication of experiments. Authors can upload supporting documentation to indicate the use of appropriate reporting guidelines for health-related research (see EQUATOR Network), life science research (see the BioSharing Information Resource), or the ARRIVE guidelines for reporting work involving animal research. Where applicable, authors should refer to any relevant reporting standards documents in this form.

If you have any questions, please consult our Journal Policies and/or contact us: editorial@elifesciences.org.

Sample-size estimation

- You should state whether an appropriate sample size was computed when the study was being designed
- You should state the statistical method of sample size computation and any required assumptions
- If no explicit power analysis was used, you should describe how you decided what sample (replicate) size (number) to use

Please outline where this information can be found within the submission (e.g., sections or figure legends), or explain why this information doesn't apply to your submission:

We used a sample size of 10 participants for the *in vivo* study. This is typical for controlled circadian/diurnal laboratory studies of human volunteers. We have previously employed n = 8 participants in a control group undergoing a similar laboratory protocol and meeting the same inclusion/exclusion criteria, to detect 24h rhythmicity of clock genes in subcutaneous adipose tissue biopsies (*1*). Given that the rhythms are often most robust for clock gene expression, and may vary between tissues, we included at least the same number of participants in this study of the entire transcriptome in skeletal muscle. In total this resulted in 60 samples to be analysed for the *in vivo* study.
For the in vitro analysis we went for a high time-resolution (2h) over two full cycles (48h) but reduced the number of donors to 2. This resulted in 100 samples to be analysed (50 samples per donor due to siCTRL and siClock condition).

Replicates

- You should report how often each experiment was performed
- You should include a definition of biological versus technical replication
- The data obtained should be provided and sufficient information should be provided to indicate the number of independent biological and/or technical replicates
- If you encountered any outliers, you should describe how these were handled
- Criteria for exclusion/inclusion of data should be clearly stated
- High-throughput sequence data should be uploaded before submission, with a private link for reviewers provided (these are available from both GEO and ArrayExpress)

eLife Sciences Publications, Ltd is a limited liability non-profit non-stock corporation incorporated in the State of Delaware, USA, with company number 5030732, and is registered in the UK with company number FC030576 and branch number BR015634 at the address 1st Floor, 24 Hills Road, Cambridge CB2 1JP | August 2014

Please outline where this information can be found within the submission (e.g., sections or figure legends), or explain why this information doesn't apply to your submission:

Both the *in vivo* and the *in vitro* experiments were performed once. Each individual participant was treated as a biological replicate. No outliers were encountered. RNA sequencing was performed only on biological replicates, no technical replicates were included in the experimental design.

Statistical reporting

- Statistical analysis methods should be described and justified
- Raw data should be presented in figures whenever informative to do so (typically when N per group is less than 10)
- For each experiment, you should identify the statistical tests used, exact values of N, definitions of center, methods of multiple test correction, and dispersion and precision measures (e.g., mean, median, SD, SEM, confidence intervals; and, for the major substantive results, a measure of effect size (e.g., Pearson's r, Cohen's d)
- Report exact p-values wherever possible alongside the summary statistics and 95% confidence intervals. These should be reported for all key questions and not only when the p-value is less than 0.05.

Please outline where this information can be found within the submission (e.g., sections or figure legends), or explain why this information doesn't apply to your submission:

The differential gene expression analysis was performed with the R package edgeR (*2*). First, transcripts expressing lower than 3 counts per million (CPM) and non-informative transcripts (e.g., non-aligned) were filtered. To minimize the log-fold changes between the samples for most genes, a set of scaling factors for the library sizes were estimated with the trimmed mean of M-values (TMM) method (*3*). The dispersion was estimated with the quantile-adjusted conditional maximum likelihood (qCML) method. Once the dispersion estimates were obtained, we performed the testing procedures for determining differential expression using the exact test (*4*). A false discovery rate (FDR) of < 0.05 was used to select differentially expressed genes.

(For large datasets, or papers with a very large number of statistical tests, you may upload a single table file with tests, Ns, etc., with reference to sections in the manuscript.)

Group allocation

- Indicate how samples were allocated into experimental groups (in the case of clinical studies, please specify allocation to treatment method); if randomization was used, please also state if restricted randomization was applied
- Indicate if masking was used during group allocation, data collection and/or data analysis

Please outline where this information can be found within the submission (e.g., sections or figure legends), or explain why this information doesn't apply to your submission:

N/A for the *in vivo* work, as all participants were grouped together.

Additional data files ("source data")

- We encourage you to upload relevant additional data files, such as numerical data that are represented as a graph in a figure, or as a summary table
- Where provided, these should be in the most useful format, and they can be uploaded as "Source data" files linked to a main figure or table
- Include model definition files including the full list of parameters used
- Include code used for data analysis (e.g., R, MatLab)

eLife Sciences Publications, Ltd is a limited liability non-profit non-stock corporation incorporated in the State of Delaware, USA, with company number 5030732, and is registered in the UK with company number FC030576 and branch number BR015634 at the address 1st Floor, 24 Hills Road, Cambridge CB2 1JP | August 2014

- Avoid stating that data files are "available upon request"

The rhythmic analysis was performed with the R software. Various reformatting functions were developed; raw data were transformed to log_2 reads per kilobase per million mapped reads (RPKM) as described previously (*5*). Only transcripts with log_2 RPKM>0 for each of the fourth conditions (2 subjects, siControl or siCLOCK) were kept, avoiding big variability for weakly expressed transcripts. The 48 time points of each condition were used to define a local regression function (loess function in R). This step allows smoothing the curve and reducing local variability. The function was then used to calculate 10 different measures (maximum and minimum slopes, first and second extremum, minimum-maximum ratio, autocorrelation, measure of scattering, residues on the loess function, residues on a linear function and period). These features were used to classify gene expression patterns in 4 different groups: rhythmic genes (category "circadian"), genes that show only one pic at the beginning of the time course (category "one peak"), linearly (category "linear") and scattered expressed genes (category "cloud"). The R package randomForest was used to classify genes. The function attributes a probability to each transcript per condition. To be classified in one category, this probability must be the highest value and superior to 0.5 in at least one category. If no probabilities were superior to 0.5 for the four categories, transcripts were grouped into model 16 (non-rhythmic). The 11 major circadian genes, including *ARNTL* (*BMAL1*), *NR1D1* (*REVERBα*), *NR1D2* (*REVERBβ*), *PER1*, *PER2*, *PER3*, *CRY1*, *CRY2*, *NPAS2*, *TEF* and *BHLHE41*, were selected to train the random forest model. The same number of genes was also integrated in the training dataset for the 3 other groups. This dataset was then passed to the random forests training algorithm and gene conditions that were assigned to one of these categories with a high score (0.9) were integrated in the training dataset. This procedure was repeated until 500 curves per group were identified.

References

1. D. T. Otway *et al.*, Rhythmic diurnal gene expression in human adipose tissue from individuals who are lean, overweight, and type 2 diabetic. *Diabetes* **60**, 1577-1581 (2011).
2. M. D. Robinson, D. J. McCarthy, G. K. Smyth, edgeR: a Bioconductor package for differential expression analysis of digital gene expression data. *Bioinformatics* **26**, 139-140 (2010).
3. M. D. Robinson, A. Oshlack, A scaling normalization method for differential expression analysis of RNA-seq data. *Genome biology* **11**, R25 (2010).
4. M. D. Robinson, G. K. Smyth, Small-sample estimation of negative binomial dispersion, with applications to SAGE data. *Biostatistics (Oxford, England)* **9**, 321-332 (2008).
5. F. Atger *et al.*, Circadian and feeding rhythms differentially affect rhythmic mRNA transcription and translation in mouse liver. *Proceedings of the National Academy of Sciences of the United States of America* **112**, E6579-6588 (2015).
